# Gender differences in cognitive reserve: implication for subjective cognitive decline in women

**DOI:** 10.1007/s10072-021-05644-x

**Published:** 2021-10-08

**Authors:** Giulia Giacomucci, Salvatore Mazzeo, Sonia Padiglioni, Silvia Bagnoli, Laura Belloni, Camilla Ferrari, Laura Bracco, Benedetta Nacmias, Sandro Sorbi, Valentina Bessi

**Affiliations:** 1 Department of Neuroscience, Psychology, Drug Research and Child Health, University of Florence, Azienda Ospedaliero-Universitaria Careggi, Largo Brambilla, 3, 50134 Florence, Italy; 2 IRCCS Fondazione Don Carlo Gnocchi, Florence, Italy; 3 Regional Referral Centre for Relational Criticalities - Tuscany Region, Florence, Italy; 4 Unit Clinic of Organizations Careggi University Hospital, Florence, Italy

**Keywords:** Subjective cognitive decline, Cognitive reserve, Sex, Gender differences

## Abstract

**Background:**

Subjective Cognitive Decline (SCD) is a self-experienced decline in cognitive capacity with normal performance on standardized cognitive tests, showing to increase risk of Alzheimer’s Disease (AD). Cognitive reserve seems to influence the progression from SCD to Mild Cognitive Impairment (MCI) and to AD. The aim of our study was to investigate gender differences in cognitive reserve evaluating how sex might modulate the role of cognitive reserve on SCD.

**Methods:**

We included 381 SCD patients who underwent clinical evaluation, neuropsychological assessment, evaluation of premorbid intelligence by the Test di Intelligenza Breve (TIB), cognitive complaints by the Memory Assessment Clinics Questionnaire (MAC-Q), and apolipoprotein E (APOE) genotyping.

**Results:**

The proportion between women and men was significantly different (68.7% [95% CI 63.9–73.4 vs 31.4%, 95% CI 26.6–36.0]). Women were younger than men at onset of SCD and at the baseline visit (*p* = 0.021), had lower years of education (*p* = 0.007), lower TIB scores (*p* < 0.001), and higher MAC-Q scores (*p* = 0.012). TIB was directly associated with age at onset of SCD in both women and men, while years of education was inversely associated with age at onset only in women. Multivariate analysis showed that sex influences TIB independently from years of education. TIB was directly associated with MAC-Q in men.

**Conclusions:**

Sex interacts with premorbid intelligence and education level in influencing the age at onset and the severity of SCD. As the effect of education was different between men and women, we speculated that education might act as a minor contributor of cognitive reserve in women.

## Introduction

Subjective Cognitive Decline (SCD) was defined as a self-experienced persistent decline of cognitive capacity in comparison with the subject’s previously normal status, during which the subject has normal age-, sex-, and education-adjusted performance on standardized cognitive tests [1]. SCD has been shown to be associated with higher neuroradiological features similar to those seen in Alzheimer’s Disease (AD) patients, such as volume loss in hippocampal/parahippocampal areas [2, 3] and amyloid deposition [4]. Two meta-analyses showed that individuals with SCD are twice as likely to develop Mild Cognitive Impairment (MCI) or dementia as individuals without [5, 6]. For these reasons, SCD is getting growing attention from clinical research as it represents a target population for the identification of individuals in the preclinical phase of AD.

SCD could also be due to non-degenerative conditions, such as normal aging, personality traits, psychiatric conditions, neurologic and medical disorders, substance use, and medication [7].

Several studies applying multifactorial approaches found that demographic and genetic factors, such as age at onset [1, 8], APOE ε4 genotype [8], and cognitive reserve [9–11], may influence the risk of progression from SCD to MCI and dementia.

Sex is a demographic variable which has been associated with AD: previous studies showed that two thirds of those diagnosed with AD are women [12]. Sex seems to have a role also on SCD prevalence, as several authors described a higher proportion of women in the SCD population [13–15] and a tendency for women to report more concerns associated with SCD than men [16, 17]. However, possible relationships between sex and SCD features have not been explored so far.

In particular, we hypothesized that sex might influence cognitive reserve [18]. Gender differences in the cognitive reserve have been previously described in AD patients, highlighting that AD women present lower education levels [18].

However, studies about cognitive reserve contributors are from mixed-sex cohorts and did not divide their samples by sex. Therefore, whether characteristics of cognitive reserve hold for both women and men is unknown [18].

To the best of our knowledge, no studies have analyzed the interaction between sex and cognitive reserve in SCD cohorts. In this scenario, we aimed to investigate gender differences in cognitive reserve evaluating how sex might modulate the role of cognitive reserve on SCD.

## Materials and methods

### Participants and clinical assessment

As part of a longitudinal, clinical–neuropsychological–genetic survey on SCD, we included 381 patients who were referred to the Centre for Alzheimer’s Disease and Adult Cognitive Disorders of Careggi Hospital in Florence between January 1994 and August 2020.

Inclusion criteria were (1) age at baseline > 40 years; (2) complaining of cognitive decline with a duration of ≥ 6 months; (3) normal functioning on the Activities of Daily Living and the Instrumental Activities of Daily Living scales [19]; and (4) unsatisfied criteria for dementia at baseline [20, 21]. Exclusion criteria were history of head injury, current neurological and/or systemic disease, symptoms of psychosis, major depression, alcoholism, or other substance abuse.

All participants underwent a comprehensive family and clinical history, general and neurological examination, extensive neuropsychological investigation, estimation of premorbid intelligence, and assessment of depression at baseline. One hundred fifty-five subjects underwent APOE genotyping. Positive family history was defined as one or more first-degree relatives with documented cognitive decline. Patients underwent clinical and neuropsychological follow-up every 12 or 24 months.

In all cases, there was a perfect correspondence between sex (the biological designation) and gender (the social construct).

The local ethics committee approved the protocol of the study. All participants gave written informed consent to participate in the study.

### Neuropsychological assessment

All subjects were evaluated by means of an extensive neuropsychological battery standardized and described in further detail elsewhere [22]. The battery consisted of global measurements (Mini-Mental State Examination), tasks exploring verbal and spatial short-term memory (Digit Span; Corsi Tapping Test), verbal long-term memory (five words and paired words acquisition; recall after 10 min; recall after 24 h; Babcock short story immediate and delayed recall), and language (token test; category fluency task) [22]. Visual–spatial abilities were also evaluated by Rey–Osterrieth complex figure copy, and visuospatial long-term memory was assessed by means of recall of Rey–Osterrieth complex figure test [23]; attention/executive function was explored by means of dual task [24], phonemic fluency test [25], and trail making test [26]. Everyday memory was assessed by means of the Rivermead Behavioral Memory Test (RBMT) [27]. All raw test scores were adjusted for age, education, and gender according to the correction factor reported in validation studies for the Italian population [22–27].

In order to estimate the premorbid intelligence, all cases were assessed at baseline by the Test di Intelligenza Breve (TIB, i.e., Brief Intelligence Test) [28], an Italian version of the National Adult Reading Test (NART) [29]. The NART is a single-word, oral reading test consisting of 50 items. All the words are irregular, that is, they violate grapheme–phoneme correspondence rules. Since Italian is a transparent language, the reading task could not be based on the irregularity in the grapheme-to-phoneme conversion as for the NART but rather on the irregularity of words with less frequent stress patterns. The presence and severity of depressive symptoms were evaluated by means of the 22-item Hamilton Depression Rating Scale (HRSD) [30]. Cognitive complaints were explored at baseline using a survey based on the Memory Assessment Clinics Questionnaire (MAC-Q) [31]. We defined the presence of cognitive complaints if participants perceived decline in cognitive capacity than in the past or if they reported difficulties in carrying out at least four of the following activities: remembering the name of a person just introduced to them; recalling telephone numbers or zip codes used on a daily or weekly basis; recalling where they put objects in their home or office; remembering specific facts from a newspaper or magazine article they just read; and remembering the item(s) they intend to buy when they arrive at the grocery store or pharmacy.

### APOE ε4 genotyping

A standard automated method (QIAcube, QIAGEN) was used to isolate DNA from peripheral blood samples. APOE genotypes were investigated by HRMA [32]. Two sets of PCR primers were designed to amplify the regions encompassing rs7412 (NC_000019.9:g.45412079C > T) and rs429358 (NC_000019.9:g.45411941 T > C). The samples with known APOE genotypes, which had been validated by DNA sequencing, were used as standard references. The APOE genotype was coded as APOE ε4 − (no APOE ε4 alleles) and APOE ε4 + (presence of one or two APOE ε4 alleles).

### Statistical analysis

Scores at cognitive tests were reported as *z* scores calculated by the mean and standard deviation (SD) with respect to the Italian general population reported in the literature for each neuropsychological test. We tested for the normality distribution of the data using the Shapiro–Wilk test. Patient groups were characterized by using means and standard deviations (SD), median and interquartile range (IQR), frequencies or percentages and 95% confidence interval (95% CI) for continuous distributed variables, continuous non-normally distributed variables, and categorical variables, respectively. We used *t* test or non-parametric Mann–Whitney *U* test for between groups’ comparisons, Pearson’s correlation coefficient or non-parametric Spearman’s ρ (rho) to evaluate correlations between groups’ numeric measures, and chi-square test to compare categorical data. We used multiple linear regression for multivariate analysis. All statistical analyses were performed with SPSS software v.25 (SPSS Inc., Chicago, USA) and R 4.0.3 (R Foundation for Statistical Computing, Vienna, 2013).

## Results

### Description of the sample and differences between genders

Demographic features, APOE ɛ4 proportion, TIB, Mini-Mental State Examination (MMSE), and MAC-Q mean values of the whole sample are summarized in Table 1. In particular, proportion of women (262, 68.7% [95% CI 63.9:73.4]) was significantly higher than men (119, 31.3% [95% CI 26.6:36.0]).

**Table 1:** Demographic features in the whole cohort and comparison between women and men

	Whole cohort (*n* = 381)	Women (*n* = 262)	Men (*n* = 119)
Women	262 (68.7%)	–	–
Men	119 (31.3%)		
Age at baseline in years	62.4 (± 8.8)	61.7 (± 9.0)*	64.0 (± 8.4)*
Age at onset in years	58.5 (± 9.2)	57.8 (± 9.5)^	60.1 (± 8.4)^
Disease duration in years	3.9 (± 3.5)	3.9 (± 3.6)	3.9 (± 3.4)
Family history of AD	53.5% [48.4–58.5]	53.2% [47.2–59.3]	53.9% [44.8–63.2]
Years of education	11.9 (± 4.4)	11.5 (± 4.5)^§^	12.9 (± 4.2)^§^
MMSE	28.1 (± 1.9)	27.9 (± 2.0)^ç^	28.4 (± 1.9)^ç^
TIB	110.7 (± 7.3)	109.0 (± 7.4)^†^	114.7 (± 5.4)^†^
HDRS	5.9 (± 4.0)	6.3 (± 4.1)^+^	5.2 (± 3.8)^+^
MAC-Q	25.9 (± 3.0)	26.3 (± 3.1)°	25.0 (± 2.7)°
APOE ɛ4 +	27.7% [20.7–34.8]	22.2% [14.4–30.0]^&^	40.4% [26.4–54.4]^&^

Values quoted in table are mean (± SD) or percentages [95% CI]. Statistically significantly different values between males and females are reported as underlined characters (significant differences at *p* < 0.05). *TIB*, Test di Intelligenza Breve; *HDRS*, Hamilton Depression Rating Scale; *MAC*-*Q*, Memory Assessment Clinics Questionnaire*.* *p = 0.021; ^*p* = 0.028; ^§^*p* = 0.007; ^ç^*p* = 0.030; ^†^*p* < 0.001; ^+^*p* = 0.009; °*p* = 0.012; ^&^*χ*^2^ = 5.4, *p* = 0.020

We compared descriptive variables between women and men and found that women had lower years of education (11.5 ± 4.5 years vs 12.90 ± 4.2 years, *p* = 0.007) and TIB scores (109.0 ± 7.4 vs 114.7 ± 5.4, *p* < 0.001) compared to men. Age at onset of SCD and age at baseline were higher in men compared to women (respectively, 60.1 ± 8.4 years vs 57.8 ± 9.5 years, *p* = 0.028; 61.7 ± 9.0 years vs 64.0 ± 8.4 years, *p* = 0.028). On the other hand, MAC-Q score was higher in women as compared to men (26.3 ± 3.1 vs 25.0 ± 2.7, *p* = 0.012). Proportion of APOE ɛ4 was 27.7% (95% CI 20.7:34.8) and was higher in men compared to women (40.4% [95% CI 26.4:54.4] vs 22.2% [95% CI 14.4:30.0], *χ*^2^ = 5.4, *p* = 0.020). HDRS was higher in women compared to men (6.3 ± 4.1 vs 5.2 ± 3.8, *p* = 0.009) (Table 1).

### Relationship between gender and cognitive reserve proxies

**Fig. 1:**
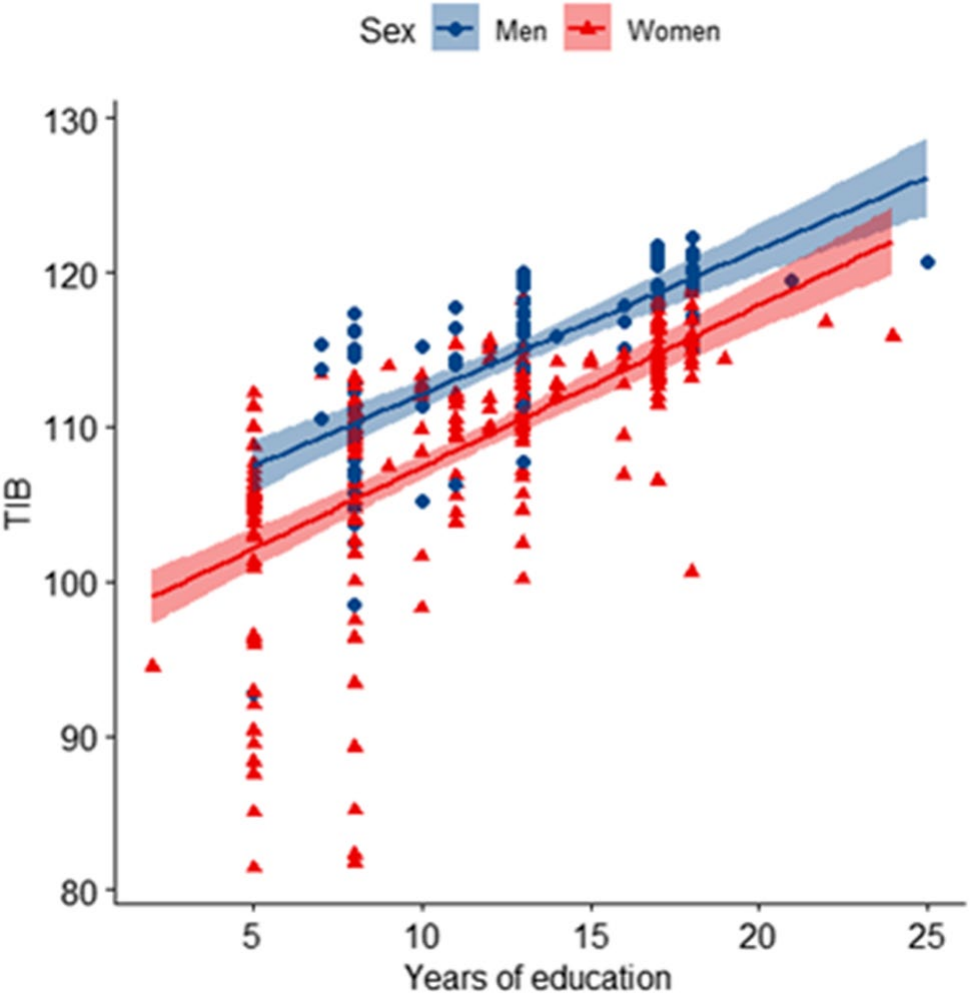
Sex difference of the correlation between premorbid intelligence and years of education. Scatter plots with lines of best fit (95% CI) show the relationship between TIB and years of education. The correlation between TIB and years of education was significant both in men (Spearman’s rho 0.753, *p* < 0.001) and in women (Spearman’s rho 0.754, *p* < 0.001)

In order to ascertain if the influence of sex on TIB was independent of other variables, we run a multiple regression analysis considering TIB as a dependent variable and sex, years of education, APOE ɛ4, and HDRS as independent covariates. The multiple regression model statistically significantly predicted TIB (*F* [4, 119] = 31.58, *p* < 0.001, adj. *R*^2^ = 0.515). Among covariates, years of education (*B* = 1.003 [95% CI 0.77:1.23], *p* < 0.001) and sex (B =  − 5.22 [95% CI − 7.53: − 2.92], *p* < 0.001) were statistically significant. Therefore, we performed the same analysis in women and men separately. We found that the relationship between years of education and TIB was still present in both groups (Table 2). Notably, the constant of the equation of men was higher than that obtained in the women subgroup (102.76 [95% CI 100.13:105.38] vs 96.91 [95% CI 94.86:98.96]) (Fig. 1).

**Table 2:** Multiple regression model for prediction of TIB

	Whole cohort		Women		Men	
	*B* (95% CI)	*β*	*B* (95% CI)	*β*	*B* (95% CI)	*β*
(Constant)	103.84 (100.08:107.59)		96.91 (94.86:98.96)		102.76 (100.13:105.38)	
Sex (F = 1, M = 0)	− 5.22*** (− 7.53: − 2.92)	− 0.30	–	–	–	–
Years of education	1.00*** (0.77:1.23)	0.57	1.04*** (0.75:1.32)	0.60	0.76*** (0.41:1.12)	0.69
APOE ɛ4 +	0.25 (− 1.91:2.43)	0.15	0.25 (− 3.43:3.95)	0.13	− 0.10 (− 2.60:2.40)	− 0.01
HDRS	− 0.24 (− 0.47: − 0.01)	− 0.13	− 0.28 (− 0.59:0.02)	− 0.15	− 0.04 (− 0.35:0.27)	− 0.04

*B*, unstandardized regression coefficient; *β*, standardized coefficient. All the covariates included in the analysis are reported. Age at onset was considered as a dependent variable. *p < 0.05, **p < 0.005, and ***p < 0.001

### Interaction between gender and cognitive reserve proxies on age at onset

On the whole sample, age at onset of SCD was inversely correlated with years of education (Spearman’s rho − 0.155, *p* = 0.003) and directly correlated with TIB (Spearman’s rho 0.165, *p* = 0.004). When we tested for correlations in women and men separately, we found that years of education were inversely correlated with age at onset in women (Spearman’s rho − 0.259, *p* < 0.001), while TIB was directly correlated with age at onset in men (Spearman’s rho 0.292, *p* = 0.005).

In order to ascertain the influence of each variable on the age at onset of SCD, we run a multiple regression analysis. We considered the age at onset of SCD as a dependent variable and sex, years of education, TIB, MMSE, HDRS, and APOE ɛ4 as covariates.

**Fig. 2:**
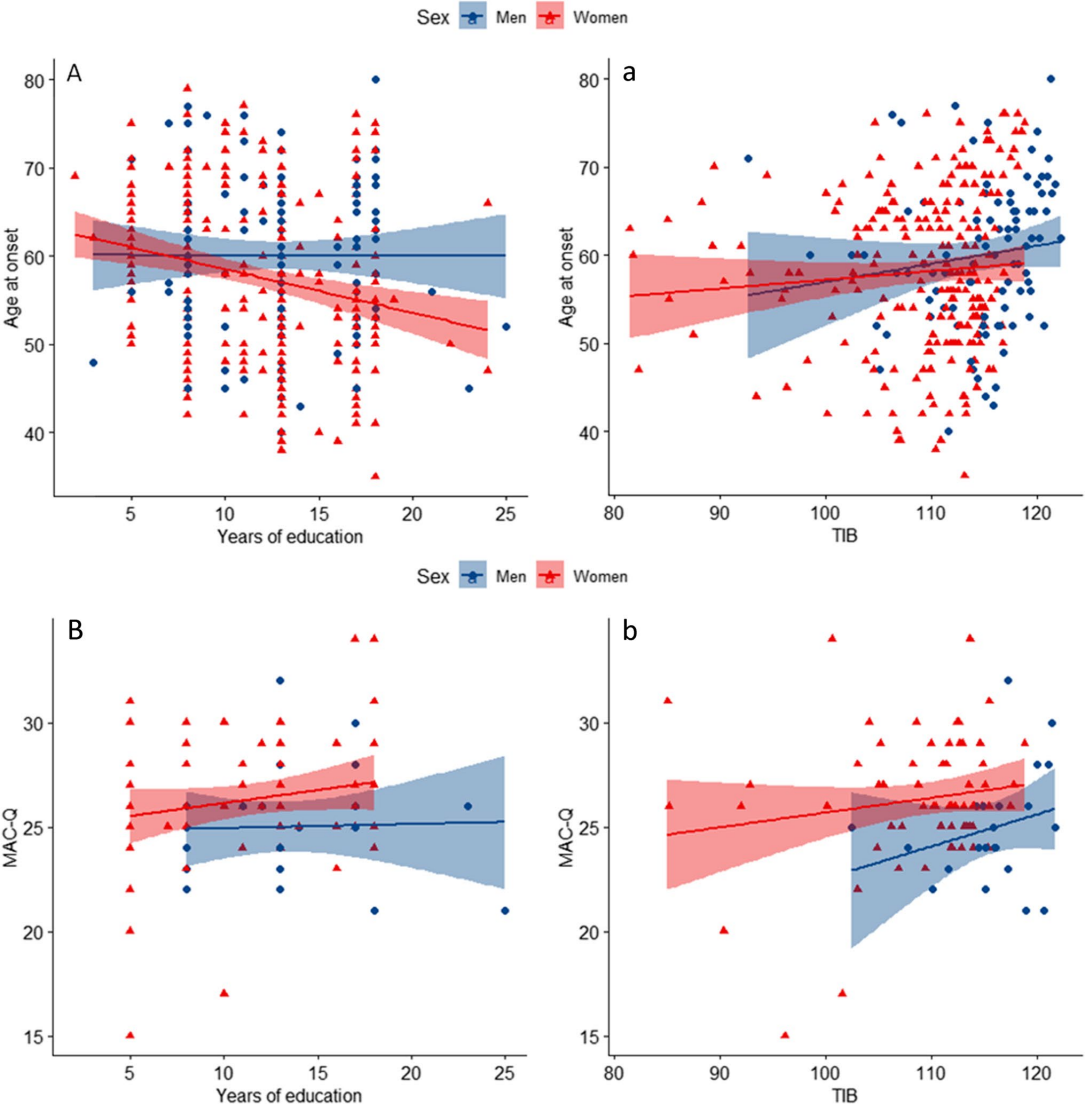
Interaction between gender and cognitive reserve proxies on age at onset of SCD and cognitive complaints. (**A**) Age at onset: opposite effect of premorbid intelligence and education in women. Scatter plots with lines of best fit (95% CI) show the relationship between age at onset and years of education (a) and TIB (b). The correlation between age at onset of SCD and years of education was significant in women (Spearman’s rho − 0.259, *p* < 0.001). The correlation between age at onset of SCD and TIB was significant in men (Spearman’s rho 0.292, *p* = 0.005). (**B**) MAC-Q: effect of premorbid intelligence in men. Scatter plots with lines of best fit (95% CI) show the relationship between MAC-Q and years of education (a) and TIB (b)

The multiple regression model significantly predicted age at onset of SCD (*F* [6, 117] = 3.98, *p* = 0.001, adj. *R*^2^ = 0.128). Among the covariates, years of education (*B* =  − 0.93 [95% CI − 1.35: − 0.51], *p* < 0.001) and TIB (*B* = 0.43 [95% CI 0.16:0.70], *p* = 0.002) were statistically significant (Table 2). In particular, age at onset was directly associated with TIB score but inversely associated with years of education. These associations still remained significant when we performed the same analysis only in women (years of education: *B* =  − 1.13 [95% CI − 1.58: − 0.68], *p* < 0.001; TIB: *B* = 0.38 [95% CI 0.10:0.65], *p* = 0.007) (Fig. 2A). In the men subgroup, only TIB was directly associated with age at onset (*B* = 1.20 [95% CI 0.24:2.16], *p* = 0.016) (Fig. 2A) (Table 3).

**Table 3:** Multiple regression model for prediction of age at onset

	Whole cohort		Women		Men	
	*B* (95% CI)	*β*	*B* (95% CI)	*β*	*B* (95% CI)	*β*
(Constant)	33.11 (2.02:64.21)		39.68 (8.59:70.77)		− 42.87 (− 137.59:51.85)	
Sex (F = 1, M = 0)	− 0.07 (− 3.69:3.55)	− 0.004	–	–	–	–
Years of education	− 0.93*** (− 1.35: − 0.51)	− 0.49	− 1.13*** (− 1.58: − 0.68)	− 0.60	− 0.55 (− 1.62:0.51)	− 0.26
TIB	0.43** (0.16:0.70)	0.39	0.38* (0.10:0.65)	0.34	1.20* (0.24:2.16)	0.63
APOE ɛ4 +	1.84 (− 1.31:5.00)	0.10	0.25 (− 3.43:3.95)	0.13	4.69 (− 1.38:10.77)	0.27
HDRS	0.05 (− 0.29:0.39)	0.026	− 0.09 (− 0.48:0.29)	− 0.49	− 0.28 (− 1.15:0.58)	− 0.15
MMSE	− 0.47 (− 1.24:0.30)	− 0.49	− 0.38 (− 1.23:0.48)	− 0.60	− 1.11 (− 2.94:0.71)	− 0.26

*B*, unstandardized regression coefficient; *β*, standardized coefficient. All the covariates included in the analysis are reported. Age at onset was considered as a dependent variable. *p < 0.05, **p < 0.005, and ***p < 0.001

### Interaction between gender and cognitive reserve proxies on cognitive complaints

Finally, we looked for factors influencing the difference in the severity of SCD between women and men. On the whole sample, there were no significant correlations between MAC-Q and years of education, TIB, HDRS, and MMSE. We run a multiple regression analysis considering MAC-Q as a dependent variable and sex, years of education, TIB, MMSE, HDRS, and APOE ɛ4 as covariates (Table 4). The multiple regression model did not statistically predicted MAC-Q (*F* [6, 75] = 1.46, *p* = 0.202, adj. *R*^2^ = 0.033). Among the covariates, sex presented a trend to significance (*B* = 1.68 [95% CI − 0.09:3.47], *p* = 0.064). Therefore, we performed a backward linear regression analysis in men and women separately. In the men group, the final model statistically predicted MAC-Q (*F* [3, 16] = 3.79, *p* = 0.031, adj. *R*^2^ = 0.416) and included TIB (*B* = 0.35 [95% CI 0.07:0.62], *p* = 0.017), MMSE (*B* =  − 0.86 [95% CI − 1.61: − 0.15], *p* = 0.020) and HDRS (*B* =  − 0.35 [95% CI − 0.66: − 0.05], *p* = 0.024). When performed only in women, none of the regression models were significant (Table 4) (Fig. 2B).

**Table 4:** Multiple regression model for prediction of MAC-Q

	Whole cohort		Women		Men	
	*B* (95% CI)	*β*	*B* (95% CI)	*β*	*B* (95% CI)	*β*
(Constant)	22.04 (5.96:38.12)		22.81 (5.44:40.19)		8.63 (− 30.69:47.96)	
Sex (F = 1, M = 0)	1.68 (− 0.09:3.47)	0.23	–	–	–	–
Years of education	0.005 (− 0.19:0.20)	0.008	0.080 (− 0.15:0.31)	0.11	Not included	− 0.10
TIB	0.09 (− 0.04:0.22)	0.213	0.062 (− 0.08:0.25)	0.144	0.35* (0.07–0.62)	0.60
APOE ɛ4 +	− 0.75 (− 2.40:0.90)	− 0.103	− 0.33 (− 2.35:1.68)	− 0.043	Not included	− 0.07
HDRS	0.072 (− 0.11:0.26)	0.088	0.19 (− 0.04:0.42)	0.21	− 0.4* (− 0.665: − 0.054)	− 0.56
MMSE	− 0.26 (− 0.64:0.11)	− 0.15	− 0.18 (− 0.62:0.25)	− 0.11	− 0.86* (− 1.561: − 1.53)	− 0.53

*B*, unstandardized regression coefficient; *β*, standardized coefficient. All the covariates included in the analysis are reported. MAC-Q was considered as a dependent variable. *p < 0.05, **p < 0.005, and ***p < 0.001

## Discussion

Our study aimed to investigate gender differences in cognitive reserve evaluating how sex might modulate the role of cognitive reserve on SCD.

First of all, in our cohort, we found a greater proportion of women compared to men, in line with previous findings in larger populations [13–15] and reflecting the well-known sex discrepancy in AD, since two thirds of those diagnosed with AD are women [12]. We also showed that all considered variables in our works were different between female and male populations, supporting previous works suggesting considering sex differences in studies on cognition and SCD [17, 33].

As a pivotal result, we focused on the difference in cognitive reserve proxies between sexes. In our cohort, premorbid intelligence (measured as TIB) and years of education were lower in women compared to men, as commonly found in the literature [18]. In particular, we found a significant gap in premorbid intelligence between women and men with the same educational level. In other words, at constant years of education, men had higher premorbid intelligence compared to women, independently from possible confounding factors.

Secondly, we found that cognitive reserve influences the age at onset of SCD in a different manner depending on the sex of patients. Indeed, age at onset was directly associated with premorbid intelligence both in men and in women. However, in women, we found an inverse relationship between years of education and age at onset of SCD.

The direct association between premorbid intelligence and age at onset of SCD may be in line with Stern’s model of cognitive reserve, which assumes that highly intelligent or educated individuals appear to be able to better cope with the presence of a neurogenerative pathology, maintaining a normal functional level for a longer time than less intelligent or educated people [9]. There are poor data on the relationship between cognitive reserve proxies and age at the onset of SCD. The majority of the studies focused on the role of cognitive reserve in mild cognitive impairment (MCI) and AD [11, 34] and on the interaction between cognitive reserve and age at onset on the progression of cognitive decline. Furthermore, previous studies on SCD showed that lower cognitive reserve is associated with greater overall memory concerns [3].

The different effect of education between women and men is a challenging issue and may be explained by a multifactorial approach. Social factors should be taken into account. Many cognitive reserve contributors are highly gendered, including education, occupation, physical activity, and social support. Most participants in studies on cognitive reserve were AD patients, and most studies on AD were conducted in the early to mid-1900s when, because of gender norms, cognitive reserve factors such as education, occupation, and physical activity were principally male [18]. Moreover, we should consider that normal education typically ends decades before old age begins. Other experiences in adulthood and old age such as social activity [35], conscientiousness [36], cognitively demanding work [37], and purpose in life [38] could influence the late-life level of cognitive activity (roughly analogous to schooling), which has been associated with the rate of cognitive change [39, 40]. This implies that influences on cognitive reserve vary over time, with recent experiences more influential than remote experiences such as schooling [41].

As a consequence, we might hypothesize that education plays a different role according to gender, probably acting as a minor contributor of cognitive reserve in women, in line with current research [41]. This could explain why years of education and premorbid intelligence are inversely related to age at onset in women in our SCD cohort, but further studies are needed to evaluate this hypothesis. However, other factors with an already known gendered effect on the brain should be taken into account.

From a biological point of view, the reported sex-specific differences may be explained, at least in part, by the role of sex hormones on brain function. Estrogen is thought to have a neuroprotective effect, and estrogen loss due to menopause might have a significant effect on cognitive decline and AD [42].

Studies also suggested an interaction between sex and APOE ε4 as the presence of this allele seems to reduce or abolish the neuroprotective effect of estrogen [43, 44]. However, in our sample, women had a lower prevalence of APOE ɛ4 compared to men, and we did not find any interaction between APOE ɛ4 and cognitive reserve.

Nevertheless, other genetic factors might be considered to explain sex differences in cognitive reserve. Sex commonly and substantially influences many facets of the human brain from ion channels to brain morphology [45]. As described in previous studies, genes important in the evolution of the human brain would be expected to have sex-specific effects [46]. Future studies including more genetic variables should be carried on in order to explore this point.

Moreover, selective differences in cognitive domain performances should be considered, as neurological bases of cognitive reserve seem to be different between women and men. In fact, it has been suggested that women present an advantage for verbal memory, which could be a form of cognitive reserve specific to females, called “memory reserve” [47].

Finally, we could speculate that the opposite effect of education on the age of onset of SCD might reflect the heterogeneity of SCD. For instance, women might be more prone than men to SCD due to non-degenerative conditions, on which education may act differently from neurodegenerative cognitive decline. We aim to explore this hypothesis in future works including AD biomarkers and follow-up data.

As a further result, we found that sex and cognitive reserve influence the severity of cognitive complaints. First of all, the severity of complaints was higher in women compared to men. This finding is in line with previous studies on SCD, which demonstrated that women concern more than men about memory loss [13, 16, 48]. In the men group, we found that the higher the cognitive reserve, the worse the complaint. This result is in contrast with previous studies reporting that less-educated individuals showed a higher grade of cognitive complaint [48, 49]. This discrepancy could be due to the different cognitive reserve proxies included in our regression model. We already discussed that premorbid intelligence and years of education might contribute differently to cognitive reserve storage. Moreover, the different recruitment method should also be taken into account: in our work, memory clinic patients were included, while previous studies are community based. This distinction has been recommended also by other authors based on the evidence that demographic and neuropsychological features are different according to the recruitment method [50].

Our work has some limitations. First, the lack of biomarkers data. As the cognitive reserve hypothesis is based on the assumption that different grade of pathology load corresponds to a different grade of cognitive decline, the estimation of grade disease by means of CSF biomarkers and functional and structural imaging may undoubtedly provide useful information. Secondly, as it is a single-center study, there may be estimator and analytical biases with regard to assessment and diagnosis procedures. Moreover, as we considered a clinic-based cohort, sampling error might be possible. Finally, due to the absence of a comparison group, we cannot describe if the relationship between cognitive reserve and sex is specific to SCD patients or if they can be detected in older adult people.

On the other hand, this study has some remarkable strengths such as the relatively large sample size and the inclusion of a great number of variables, among which genetic variables, scale for depression, two cognitive reserve proxies, and a cognitive complaints measure. Moreover, to the best of our knowledge, this is one of the first studies investigating gender differences in cognitive reserve in SCD.

In conclusion, we showed that sex and cognitive reserve interact in influencing age at onset and severity of SCD. This interaction seems to hide a high degree of complexity and, at the state of the art, it is not possible to define a complete and uniform model describing the relationship between sex and cognitive reserve in SCD. The understanding of such gendered differences and complexities is pivotal for integrating cognitive reserve into a personalized medicine approach, especially in SCD cohorts’ studies, in order to better define the risk of progression to AD and to detect candidates for future treatment options.

## Abbreviations

SCD: Subjective Cognitive Decline
MCI: Mild Cognitive Impairment
AD: Alzheimer’s Disease
TIB: Test di Intelligenza Breve
HDRS: Hamilton Depression Rating Scale
